# Draft genome of the endemic alpine ground beetle *Carabus* (*Platycarabus*) *depressus* (Coleoptera: Carabidae) from long-read sequencing of a frozen archived specimen

**DOI:** 10.1093/g3journal/jkaf027

**Published:** 2025-02-24

**Authors:** Jérémy Gauthier, Cody Raul Cardenas, Matilde Nari, Conrad P D T Gillett, Emmanuel F A Toussaint

**Affiliations:** Natural History Museum of Geneva, Route de Malagnou 1, Genève 1208, Switzerland; Naturéum—State Museum of Natural Sciences, Place de la Riponne, Palais de Rumine 6, Lausanne 1005, Switzerland; Natural History Museum of Geneva, Route de Malagnou 1, Genève 1208, Switzerland; Natural History Museum of Geneva, Route de Malagnou 1, Genève 1208, Switzerland; Finnish Museum of Natural History (LUOMUS), Pohjoinen Rautatiekatu 13, Helsinki 00100, Finland; Natural History Museum of Geneva, Route de Malagnou 1, Genève 1208, Switzerland

**Keywords:** degraded DNA, long-read genome sequencing, reference genomes, Nanopore MinION technology, genome assembly

## Abstract

The rapid advancement of genomic technologies has enabled the production of highly contiguous reference genomes for nonmodel organisms. However, these methods often require exceptionally fresh material containing unfragmented high-molecular-weight nucleic acids. Researchers who preserve field-collected specimens in ethanol at ambient temperatures, prior to transferring them to long-term frozen archives, face challenges in applying advanced genomic approaches due to DNA and RNA fragmentation under suboptimal preservation conditions. To explore the potential of such preserved specimens as sources of reference genomes, we utilized Nanopore MinION technology to generate genomic data from a frozen archived specimen of the endemic alpine ground beetle *Carabus (Platycarabus) depressus*. Using a rapid in-house protocol for high-molecular-weight DNA extraction, followed by sequencing on a single flow cell, we produced 8.75 million raw reads with an N50 of 2.8 kb. The resulting assembly achieved remarkable completeness, recovering up to 98% of Benchmarking Universal Single-Copy Orthologs genes, despite a moderate N50 of 945 kb. This genome is only the second available for the taxonomically diverse genus *Carabus*, demonstrating the feasibility of using short-to-long-read sequencing on frozen archived specimens commonly housed in natural history collections. These findings open new avenues for advancing nonmodel organism genomics and its downstream applications.

## Introduction

Over the past decade, whole-genome sequencing has advanced significantly, particularly in the development of “reference” genomes that are used as benchmarks for genomic analysis (Formenti *et al*. 2022). These reference genomes have been crucial in advancing our understanding of evolutionary relationships, biodiversity (e.g. Theissinger *et al*. 2023; Zhang *et al*. 2024), and the conservation of threatened species (e.g. Brandies *et al*. 2019). However, the prioritization of species to be sequenced is often guided by criteria that do not necessarily reflect the species richness of taxonomic groups. For example, despite being the second most species-rich lineage within the order Coleoptera, the beetle suborder Adephaga—comprising over 45,000 described species—has relatively few available reference genomes (Beutel *et al*. 2020). Within this suborder, the Carabidae is one of the largest families, comprising ca. 40,000 described species (“Carabcat database” 2021). However, only 20 reference genomes are available for this highly diversified clade (Feron and Waterhouse 2022, last accessed 2024 October 1).

The recent increase in the number of available reference genomes and the continuing improvement in their quality are primarily due to the transition from short-read to long-read sequencing technologies (van Dijk *et al*. 2023). These latter technologies, such as Pacific Biosciences single-molecule real-time sequencing (hereafter abbreviated as PacBio) (Rhoads and Au 2015) and Oxford Nanopore sequencing (Lu *et al*. 2016), enable the acquisition of long sequences, significantly simplifying genome assembly and enhancing the accuracy and quality of reference genomes (van Dijk *et al*. 2023). However, the ability to sequence long fragments is constrained by the quality of the initial source material, especially the length of DNA fragments obtained during extraction. Moreover, certain methods require a significant size selection step during library preparation (e.g. PacBio). Further compounding this, the complex logistics involved in effectively preserving specimen samples in the field make it challenging to obtain samples of adequate quality, which often restricts the use of long-read technologies to a limited number of organisms. An underexplored avenue is the exploitation of preexisting frozen archived specimens, originally collected and preserved in ethanol without the expectation of serving as source material for long-read sequencing. Such specimens are typically stored in museums and laboratories in commercial freezers at −20°C for extended periods, ranging from months to years. Freezing slows down the degradation of DNA, allowing the preservation of moderately sized DNA fragments over time. Consequently, such specimens constitute a valuable yet underutilized resource for generating medium- to long-read genomic data, which is essential for assembling new high-quality reference genomes.

**Fig. 1.**
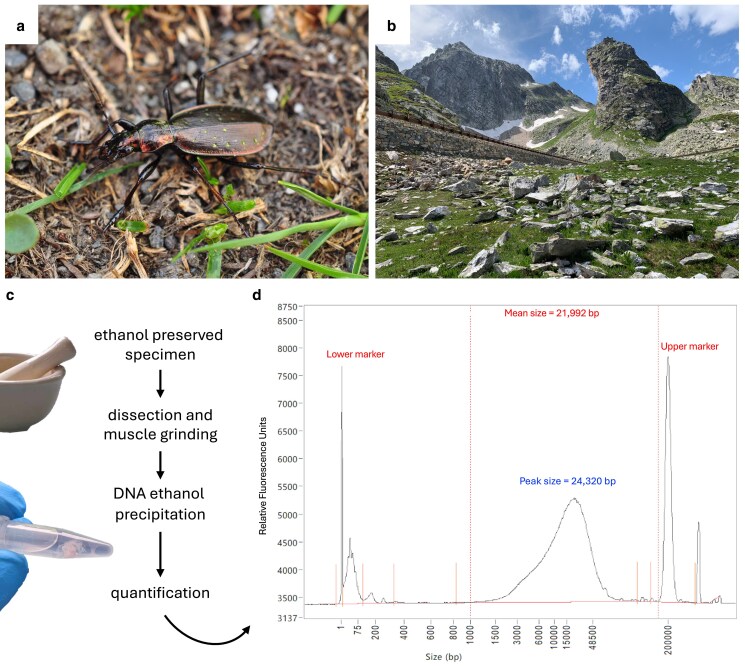
a) In situ photograph of *C. (P.) depressus* (credit: Conrad Gillett). b) In situ photograph of the habitat (credit: Emmanuel Toussaint). c) Simplified schematic representation of the DNA extraction process. d) Fragment size distribution plot obtained from a Fragment Analyzer.

To assess the potential of archived specimens for generating high-quality genomes, we leveraged the flexibility of the Oxford Nanopore MinION third-generation polynucleotide sequencer capable of sequencing both short and long DNA fragments. We employed this technology to sequence a frozen archived specimen of the giant ground beetle species *Carabus* (*Platycarabus*) *depressus* (Bonelli, 1810). This predatory species is endemic to the Alps, where it is widely distributed across most of its arc, from the Ligurian and Maritime Alps in the West, to the Julian Alps in the East. It is present in France, Italy, Switzerland, and Austria (Pauli *et al*. 2024), inhabiting an elevational range between ∼1,000 and 2,500 m that encompasses its typical habitat of high alpine meadows in addition to lower elevation woodlands, where it can be a dominant species (Turin *et al*. 2003) (Fig. 1, a and b).

Our objective was to test the potential of such intermediate-quality samples, commonly represented today in natural history collections, to generate useful genome-scale data. Specifically, we aimed to extract high-molecular-weight DNA from a single specimen of *C. depressus* that had been preserved at −20°C in 96% ethanol for an extended period of time (more than 4 years). Using an in-house protocol, we sought to assemble a draft genome from this archived material.

## Material and methods

### High-molecular-weight DNA extraction and Nanopore sequencing

A single male specimen of *C. depressus* was collected under a large stone in an alpine meadow at the Colle del Gran San Bernardo, Piemonte, Italy, in July 2019 (Fig. 1, a and b). The specimen was fixed in a 30-mL Starstedt (Nümbrecht, Germany) tube containing 96% EtOH before being transported at ambient temperature to the lab, where it was stored at −20°C until a high-molecular-weight DNA extraction was performed in February 2024. To obtain source tissue, we removed thoracic and abdominal tissue (avoiding the fore and hind gut) and a posterior leg from the specimen (representing an approximate volume of 0.01 cm^3^), while it was submerged in 96% EtOH. Following extraction, the specimen was mounted and is deposited in the Natural History Museum of Geneva collection with voucher code CBX1139. We relied on an in-house protocol largely inspired from the one recently described in the study by Lafon *et al*. (2024). The dissected tissue was first dried and then suspended in 500 μL of Qiagen Blood and Tissue Kit Lysis buffer (Qiagen, Hilden, Germany). After using a pestle and mortar to homogenize the tissue, we added proteinase K at 10% of the volume of the lysis buffer and RNase A at 2% of this volume (Fig. 1c). The final lysis solution was incubated in an Eppendorf thermomixer at 56°C with 300 rpm agitation for 1 h. Postincubation, the lysis solution was centrifuged at 11,000 g at room temperature for 10 min, after which we added 3 M sodium acetate at 10% of the volume of the eluted supernatant. After gently mixing the solution, we precipitated the DNA using 96% EtOH at twice the volume of the lysis solution (Fig. 1c). The eluted DNA was then washed with 70% EtOH twice and incubated at 37°C for 1 h. The DNA quantification was performed using a Qubit Broad Range kit (Thermo Fisher Scientific) and High Sensitivity Large Fragment 50 kb (DNF-464-33) on an Agilent 5200 Fragment Analyzer (Agilent, Santa Clara, CA, USA). Finally, the DNA template was cleaned using 1× Ampure magnetic beads (SPRI technology) and requantified. The final library was prepared using the Oxford Nanopore Ligation Sequencing Kit V14 (SQK-LSK114, Oxford Nanopore) following the manufacturer's protocol for sequencing large genomic fragments. Sequencing was performed on a MinION Mk1C and R10.4.1 Flow Cell (FLO-MIN114, Oxford Nanopore) for 45 h with a minimum read length of 200 bp, and base calling was performed with the fast model on the Mk1C device.

### Genome assembly and evaluation

Base calling was performed on raw sequencing data using Dorado v0.3.1 (https://github.com/nanoporetech/dorado) with the “dna_r10.4.1_e8.2_400bps_sup@v4.2.0” configuration corresponding to the flow cell used for the sequencing. Base calling was performed on the Baobab HPC service of the University of Geneva using GPUs. Raw bam files were converted to fastq using samtools sort v1.4 (Li *et al*. 2009) and then bedtools bamtofastq (Quinlan and Hall 2010). We used seqkit rmdup -n -D (Shen *et al*. 2016) to remove duplicate sequences by name before assembly. The processed reads were then assembled using flye2 v2.9.3 (Kolmogorov *et al*. 2019) with the default settings for corrected Nanopore reads. The evaluation of the genome was performed following BlobTools2 guidelines v2 (Laetsch and Blaxter 2017; Challis *et al*. 2020). To identify putative contamination, we performed BLAST using blastn v2.12 (Camacho *et al*. 2009) with the NCBI nucleotide database and diamond v2.1.8 (Buchfink *et al*. 2015) with the Universal Protein resource (UniProt, 2024_2 release) (UniProt Consortium 2023). Exclusion criteria were based on the blast and diamond results in blobtools using the best sum order of the phylum for any nonarthropod or “no-hit” matches. Fastq reads were mapped against the contigs using minimap2 (Li 2018, 2021) with the map-ont option. Coverage was calculated using bedtools (Quinlan and Hall 2010). Benchmarking Universal Single-Copy Orthologs (BUSCO) genes were identified using the insecta_odb10 (Seppey *et al*. 2019). The final contig assembly was filtered with a minimum contig length of 1 kb, 30% GC content, and a minimum coverage of 20×. Finally, mtDNA contigs were identified using blastn with the maximum target sequences set to 20, a maximum of 1 query–subject pair, and E-values exceeding 1e−25.

### Genome annotation

Protein-coding genes were predicted using BRAKER3 (Gabriel *et al*. 2024) following a dedicated long-read protocol and integrating evidence from RNA-seq and protein data. First, RNA-seq data obtained from different life stages of *C.* (*Ohomopterus*) *uenoi* (Ishikawa, 1960), i.e. 3rd instar larvae, male pupae, and female pupae, sequenced using a 454 GS FLX Titanium by Fujimaki *et al*. (2014), were assembled using SPAdes v3.13 (Bankevich *et al*. 2012) and –rna mode. The resulting transcripts were mapped onto the genome using minimap2 and splice:hq option (Li 2018, 2021). Gene predictions were generated using GeneMarkS-T (Tang *et al*. 2015). Second, reference proteins were extracted from OrthoDB v11 (Kuznetsov *et al*. 2023) to retain Insecta data, which were subsequently used in the BRAKER2 pipeline to design hints combining diamond (Buchfink *et al*. 2021), spaln2 (Iwata and Gotoh 2012), GeneMark_ES (Lomsadze *et al*. 2005), Prothint (Brůna *et al*. 2020), and Augustus (Hoff and Stanke 2019). Finally, the 2 sets of predictions were combined using TSEBRA (Gabriel *et al*. 2021). Proteins were extracted from the annotation, and their completeness was evaluated using BUSCO under protein mode (Seppey *et al*. 2019).

### Phylogenetic inferences

BUSCO genes identified in all published genomes, along with those recovered for *C. depressus*, were used to reconstruct the phylogenetic relationships. This analysis incorporated the newly sequenced species and existing Carabidae genomes obtained from the Arthropoda Assembly Assessment Catalogue (Feron and Waterhouse 2022). The resulting genes were aligned with MAFFT v7.505 (Katoh and Standley 2013) with –auto option and trimmed using trimAl v1.4 (Capella-Gutiérrez *et al*. 2009). Matrices were generated using AMAS (Borowiec 2016). A concatenated maximum likelihood (ML) tree was constructed using IQTREE2 (Minh *et al*. 2020) with automated model selection in ModelFinder (Kalyaanamoorthy *et al*. 2017), chosen according to BIC. We selected *Liopterus haemorrhoidalis* (Fabricius, 1787) (Dytiscidae) as an outgroup for ML analysis. Branch support was assessed using 1,000 replicates of Shimodaira–Hasegawa approximate likelihood ratio test (SH-aLRT) and 1,000 replicates of ultrafast bootstrap (UFBoot).

## Results and discussion

### DNA extraction, sequencing, and assembly statistics

The newly developed room temperature extraction protocol used in this study yielded sufficient, relatively high-molecular-weight DNA to allow for successful subsequent long-read sequencing. This protocol requires less extensive laboratory infrastructure and logistics compared with other methods for extracting high-molecular-weight DNA, which, for example, often require the use of liquid nitrogen (Brown and Coleman 2019). By directly precipitating DNA using ethanol, our extraction protocol offers a more cost-effective and rapid alternative to commercial kits (also see Lafon *et al*. 2024). The extraction resulted in 1.085 µg of DNA and eluted in 100 µL, with a Qubit concentration of 11.8 ng/µL. Despite having been stored under suboptimal conditions for over 4 years, the DNA fragment size distribution of the specimen showed a normal distribution with a mean fragment length of 24.3 kb (Fig. 1d). To maximize the DNA yield for sequencing, including both short and long fragments, purification was performed using beads (1×) without size selection. This yielded a total of 543.6 ng (40.3 fmol) of genomic DNA with a Qubit concentration of 6.04 ng/μL, available for library preparation.

**Fig. 2.**
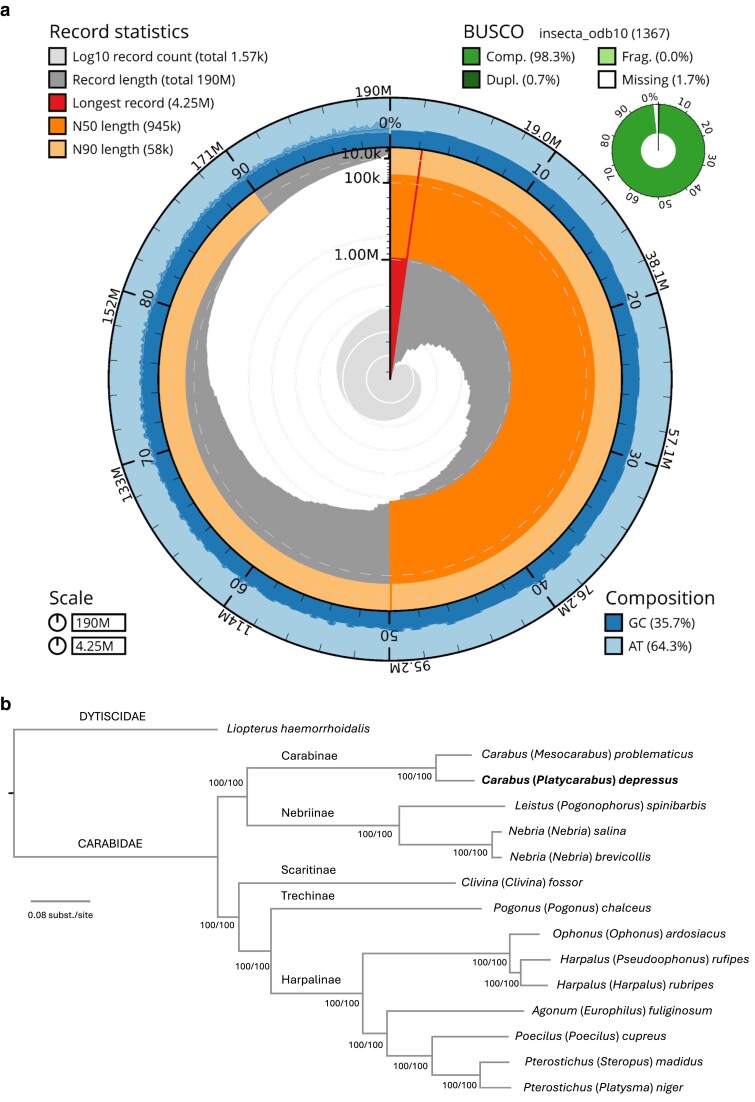
a) Snail plot of *C. (P.) depressus* genome showing the contig size distribution. Descriptive statistics including total length, N50, and N90 length are indicated in the up-left corner. BUSCO scores are in the up-right corner, and GC content is in the down-left corner. b) Phylogenetic placement of *C.* (*P.*) *depressus* genome using BUSCO genes. Branch support is indicated using SH-aLRT and UFBoot.

Although less than half the recommended input of high-molecular-weight gDNA was used for library preparation, a total of 8.75 million reads were generated with the Oxford Nanopore Mk1c device, achieving an N50 of 2.8 kb. This reduced amount of DNA may explain the premature end of the sequencing process and a lower yield than that announced by the provider. After base calling with dorado, 6.4 million reads with an N50 of 3.0 kb were obtained and assembled into a draft genome composed of 3,665 contigs with a total length of 199 Mb, an N50 of 773 kb, and a mean coverage of 41× (Supplementary Table 1). Curation of the genome, based on coverage, GC content, and BLAST results, resulted in 1,569 contigs with a total length of 190 Mb, coverage of 43×, and an N50 of 945 kb (Fig. 2a; Supplementary Fig. 1). In total, 7.98 Mb from 1,683 contigs representing potential contaminant sequences were removed along with the mtDNA contigs from the final nuclear genome assembly. Despite its level of fragmentation into numerous contigs, the completeness estimate using BUSCO indicated that the genome is complete (98.3%) and has no fragmented BUSCO genes (0.0%). The only other existing genome of a congeneric species, *C.* (*Mesocarabus*) *problematicus* (Herbst, 1786) (GCA_963422195.1), was sequenced using PacBio technology from a specimen preserved in liquid nitrogen immediately preceding extraction at the sequencing facility. Such ideal preservation conditions are understandably difficult to replicate with nonmodel and/or nonlocal organisms. This latter genome is composed of 222 scaffolds having a total size of 254 Mb, an N50 of 17.5 Mb, and a BUSCO completeness of 99.2%. In comparison with the limited existing genomic resources for the Adephaga clade, the descriptive statistics for the newly generated *C. depressus* genome highlight its value as a draft reference genome. This genome is suitable for population-scale resequencing studies (Formenti *et al*. 2022). Additionally, its completeness and gene annotations make it well suited for comparative genomic analyses (Dunn and Munro 2016).

### BUSCO phylogenomic inference

A median of 1,334 BUSCO genes were recovered from the different targeted Adephaga genomes (min. 1,260, max. 1,360). The final alignment used for phylogenetic inference was composed of 1,367 BUSCO genes and contained 853,423 parsimony-informative sites. ModelFinder recovered 104 partitions for ML phylogenetic inference. The resulting phylogenetic tree is robust, with maximal branch support (SH-aLRT = 100/UFBoot = 100) across the topology (Fig. 2b). As expected, we recover the newly sequenced genome of *C. depressus* as sister to that of *C. problematicus*, thereby forming a monophyletic genus *Carabus*. Where multiple representative samples were included, we also recovered all subfamilies of Carabidae as monophyletic. Notably, the subfamily Nebriinae was recovered as sister to Carabinae—a result consistent with that of the study by Vasilikopoulos *et al*. (2021). Despite the limited taxon sampling in this study, our robust phylogenomic inference based on reference genomes is consistent with recent studies. The new reference genome is a significant contribution to advancing our understanding of ground beetle evolution.

## Gene structural annotation

The structural annotation performed using BRAKER3 and combining hints from Coleoptera proteins and RNA-seq data resulted in the annotation of 17,224 genes (Supplementary Table 1). The completeness evaluation performed using BUSCO on the resulting proteins identified 87.6% of complete genes (84.4% of single-copy and 3.2% of duplicated). The number of annotated genes is consistent with the literature: according to data extracted from the Arthropoda Assembly Assessment Catalogue, among the 50 existing annotations of coleopteran genomes, the average number of genes identified is 20,005 (SD = 7,872) (Feron and Waterhouse 2022).

## Supplementary Material

jkaf027_Supplementary_Data

## Data Availability

Genome assembly and annotation have been made available in the NCBI under JBLKVO000000000 accession number. The annotation is also available at GSA Figshare: https://doi.org/10.25387/g3.28652057. Raw genomic data can be found under NCBI BioProjects PRJNA1171461. The assembly and annotation pipelines, including custom scripts, have been made available in the Github repository: https://github.com/crcardenas/CBX1139_genome. Supplemental material available at G3 online.
