# Supplementary material for: Draft genome of the endemic alpine ground beetle Carabus (Platycarabus) depressus (Coleoptera: Carabidae) from long-read sequencing of a frozen archived specimen
Source: G3 (Bethesda). 2025 Feb 24;15(5):jkaf027. doi: 10.1093/g3journal/jkaf027 (PMC12060234; doi:10.1093/g3journal/jkaf027)

Supplementary Materials for

Draft genome of the endemic alpine ground beetle *Carabus* (*Platycarabus*) *depressus* (Coleoptera: Carabidae) from long-read sequencing of a frozen archived specimen

Jérémy Gauthier[1,2,†,*], Cody Raul Cardenas[1,†], Matilde Nari[1], Conrad P.D.T. Gillett[3], Emmanuel F.A. Toussaint[1]

[1] *Natural History Museum of Geneva, Route de Malagnou 1, 1208 Genève, Switzerland*

[2] *Naturéum - State Museum of Natural Sciences, Place de la Riponne, Palais de Rumine 6, 1005 Lausanne, Switzerland*

[3] *Finnish Museum of Natural History (LUOMUS), Pohjoinen Rautatiekatu 13, 00100 Helsinki, Finland*

Supplementary Table 1. Summary statistics for the genome sequencing, assembly and annotation of *C. depressus*.

	C. depressus
**Oxford Nanopore data**	
Reads	6,453,688
Average size (bp)	1463.1
N50 (bp)	3044
Largest (bp)	81,136
Total length (bp)	9,442,364,531
**Assembly**	
Number of contigs	1569
Average size (bp)	121,363.32
N50 (bp)	945,386
N90 (bp)	57,976
Largest (bp)	4,253,149
Total length (bp)	190,419,055
BUSCO	C:98.3%[S:97.5%,D:0.7%],F:0.0%,M:1.7%,n:1367
**Annotation**	
Protein-coding genes	17,224
Average gene size (bp)	412.51
BUSCO	C:87.6%[S:84.4%,D:3.2%],F:1.0%,M:11.4%,n:1367

Supplementary Figure 1. Genome content before (**A**) and after (**B**) decontamination using BlobTools. The plot shows variation in GC (guanine+cytosine) proportion (x-axis), mapped read coverage (y-axis), and blast-classification of the assembly scaffolds, from which putative contaminants are commonly identified as outliers of the distributions.

A

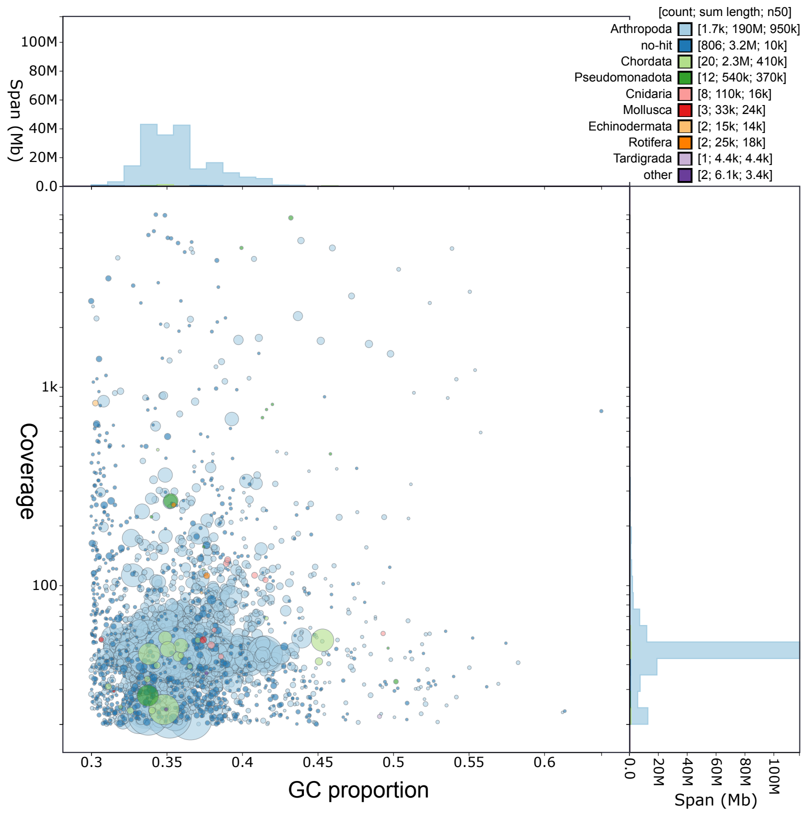

B

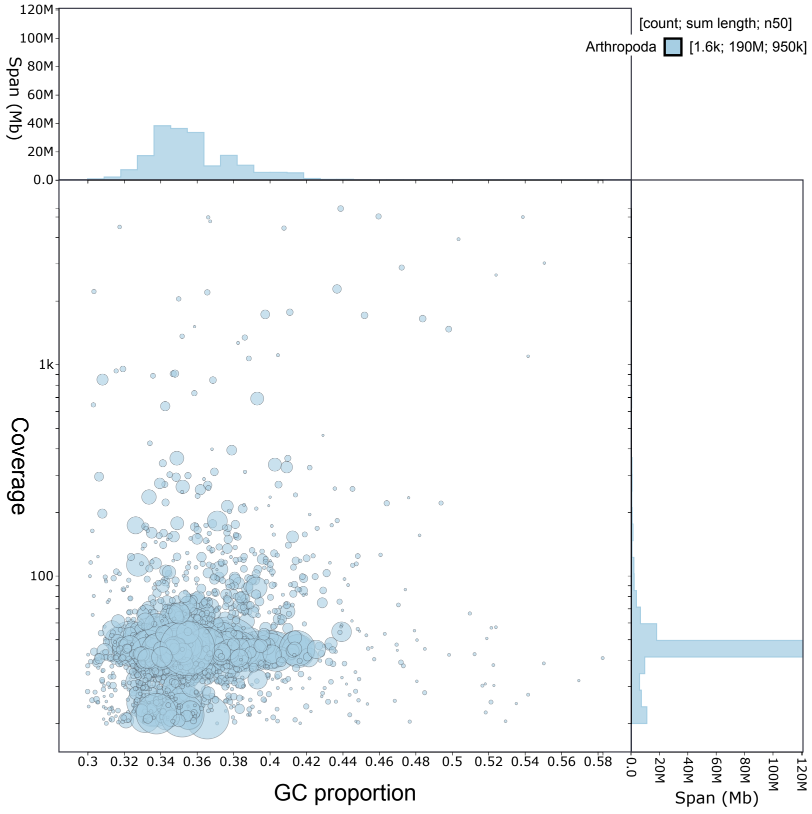

Supplement: jkaf027_Supplementary_Data [file jkaf027_supplementary_data.pdf]
